# Water Soluble Usnic Acid-Polyacrylamide Complexes with Enhanced Antimicrobial Activity against *Staphylococcus epidermidis*

**DOI:** 10.3390/ijms14047356

**Published:** 2013-04-02

**Authors:** Iolanda Francolini, Vincenzo Taresco, Fernanda Crisante, Andrea Martinelli, Lucio D’Ilario, Antonella Piozzi

**Affiliations:** Department of Chemistry, “Sapienza” University of Rome, Piazzale A. Moro 5, Rome 00185, Italy; E-Mails: iolanda.francolini@uniroma1.it (I.F.); vincenzo.taresco@uniroma1.it (V.T.); fernanda.crisante@uniroma1.it (F.C.); andrea.martinelli@uniroma1.it (A.M.); lucio.dilario@uniroma1.it (L.D.)

**Keywords:** usnic acid, polyacrylamide, antimicrobial activity, drug delivery, solubility

## Abstract

Usnic acid, a potent antimicrobial and anticancer agent, poorly soluble in water, was complexed to novel antimicrobial polyacrylamides by establishment of strong acidic-base interactions. Thermal and spectroscopic analysis evidenced a molecular dispersion of the drug in the polymers and a complete drug/polymer miscibility for all the tested compositions. The polymer/drug complexes promptly dissolved in water and possessed a greater antimicrobial activity against *Staphylococcus epidermidis* than both the free drug and the polymer alone. The best results were obtained with the complex based on the lowest molecular weight polymer and containing a low drug content. Such a complex showed a larger inhibition zone of bacterial growth and a lower minimum inhibitory concentration (MIC) with respect to usnic acid alone. This improved killing effect is presumably due to the reduced size of the complexes that allows an efficient cellular uptake of the antimicrobial complexes. The killing effect extent seems to be not significantly dependent on usnic acid content in the samples.

## 1. Introduction

Drug oral bioavailability can be reduced by several physiological factors, such as metabolic degradation prior to absorption, poor absorption from gastrointestinal tract or first-pass metabolism [1]. Low solubility and subsequent unsatisfactory dissolution rate often compromise the development of new active molecules, hindering their usage in clinics.

In an attempt to improve drug oral bioavailability, various pharmaceutical formulation technologies have been developed, including particle size reduction, use of surfactants or complex formation.

Usnic acid, one of the most extensively studied lichen derivates, is known to possess several interesting biological properties [2]. It has a good antimicrobial activity against planktonic Gram-positive bacteria [3,4], such as *Staphylococcus epidermidis*, *S. aureus*, *Enterococcus faecalis*, *Mycobacterium tuberculosis* and some pathogenic fungi [5]. Usnic acid was also recently shown to be able to prevent biofilm formation by *Staphylococcus spp.* when incorporated in polyurethanes [6,7] or in bone cements [8]. Therefore, this drug is under investigation for the prevention of medical device-related infections as an antimicrobial agent to be adsorbed on the surface of medical devices as an alternative to conventional antibiotics [9].

Besides the antimicrobial activity, usnic acid also possesses anticancer properties. Its antiproliferative activity was first reported against lung carcinoma [10] and then demonstrated against a wide variety of human cancer cell lines [11,12]. A recent study showed that usnic acid works in a p53-indipendent manner [13], making it a potential candidate for novel therapies.

Despite these recognized features, usnic acid therapeutic application has not yet been introduced, due to its low water solubility [10] and high hepatotoxicity [14,15]. Thus, its use in a safe and efficient manner requires the development of suitable dosage forms able to improve its therapeutic index.

Natural or synthetic water-soluble polymers have been extensively investigated as carriers either to increase solubility of drugs [16] or to control and target their release to specific cells [17,18]. The potential advantage of the use of synthetic polymers over the natural ones relies on the versatility of the macromolecular chemistry, that allows one tailoring the polymer molecular weight, controlling its composition and introducing specific functional groups or bioresponsive elements.

A number of polymer/anticancer drug conjugates are being tested clinically, most of which have *N*-(2-hydroxypropyl)methacrylamide copolymers as carriers [19]. So far, only a few studies concern the development of usnic acid/polymer systems. Particularly, PEG 400 and polypropylene glycol were used as co-solvents to increase usnic acid solubility [20]. However, only a poor or no effect on drug effectiveness was achieved. Better results were instead obtained by drug complexation with 2-hydroxypropyl-β-cyclodextrin in terms of both solubility and anti-proliferative activity [20]. The entrapping of a usnic acid/β-cyclodextrin complex in liposomes allowed the development of a long-term controlled-release system [21]. However, this system did not show better antimicrobial activity with respect to the free usnic acid when tested against Gram-positive bacteria.

The injection of usnic acid-loaded poly(d,l-lactic acid-co-glycolic acid) nanoparticles in Swiss mice produced a 26% increase in tumor inhibition and a reduction in drug hepatotoxicity [22], as a consequence of drug targeting.

In a more recent study [23], usnic acid was derivatized with various amine moieties, including the three natural low molecular weight polyamines, putrescine, spermidine and spermine, in order to improve drug cellular uptake by targeting the polyamine transport system (PTS). Although results showed an increase in drug cytotoxicity against human cancer cell lines, drug targeting to the PTS was unsuccessful. The authors explained the failure by an unfavorable architecture of the conjugates.

Therefore, since usnic acid encapsulation or conjugation with polymers can enhance its bioavailability, reducing its toxicity, we wanted to develop water soluble cationic polymers as drug carriers possessing a high affinity with the drug thanks to acidic-base interactions.

It is known that cationic polymers have good antimicrobial features thanks to the presence of pendant charged groups, such as phosphonium, biguanide or nitrogen groups. Different factors affect their activity, such as polymer molecular weight, the type of counterion and the alkyl chain length attached to charged group [24,25]. Although synthetic cationic polymers can be an alternative to the use of low molecular weight antimicrobials, they suffer from cytotoxicity if considered for therapeutic applications [26].

In fact, in spite of the positive charges facilitating the polymer cellular internalization, the non-specific electrostatic interactions with blood components led to undesired side effects. It was seen that the complexation of cationic polymers with biomolecules, such as DNA, leading to the partial neutralization of charges, reduces the toxicity of such polymers [27].

A widely investigated polyacrylamide in the literature is the poly-*N*-[2-(*N*,*N*-dimethylamino) ethyl]acrylamide containing charged tertiary amine groups bearing methyl residues [24,28].

In this work, an ethylacrylic amide containing charged tertiary amine groups bearing ethyl residues was first synthesized. We chose to introduce longer alkyl residues to enhance the polymer hydrophobicity and then improve its interaction with the bacterial membrane. Pursuing this strategy, propyl and butylacryl amides, always bearing ethyl residues on the charged amine groups, were also synthesized.

The water-soluble monomers were used for radical polymerization in order to obtain polymers to be employed as carriers for usnic acid. Differently from the other systems developed in the literature, such cationic polymers can establish multipoint acid-base interactions with the drug phenolic groups. These interactions could improve not only the drug bioavailability, but also polymer antimicrobial activity, as well as lower polymer toxicity by decreasing charge density.

Finally, since one of the main issue related to the use of antimicrobial agents is the development of drug resistant bacteria, the employment of an intrinsically antimicrobial carrier rather than an inert polymer could address this problem by exerting a synergistic effect with the drug in bacterial killing. Indeed, the most pursued strategy to reduce the drug resistance is the combined use of antimicrobial agents having different mechanisms of action. In our case, the cationic polymers adsorb on the negative charged bacterial membrane provoking cell disruption, while the usnic acid inhibits bacterial metabolic functions. The developed polymer/usnic acid systems were characterized by thermal and spectroscopic analysis and tested against *Staphylococcus epidermidis*.

## 2. Results and Discussion

Infectious disease is a critically important healthcare issue. The extensive use of antibiotics to eradicate the infections has contributed to the emergence of multidrug-resistant pathogens [29,30]. In the last few years, the need for suitable alternatives to replace or support systemic therapies has promoted both the use of antibiotic combinations and the development of new classes of antimicrobial compounds [24,25,31]. In this regard, antimicrobial polymers offer the promise for enhancing the efficacy of existing antimicrobial agents by increasing their efficiency and selectivity [24].

Cationic polymers based on ammonium salts are undoubtedly the most investigated macromolecular antimicrobial compounds. Similarly to host defense peptides [25], cationic polymers accumulate at the level of the polyanionic bacterial cell surface. They have a broad-spectrum of activity, since both Gram-negative and Gram-positive bacteria display on their surfaces anionic charges.

In this study, a hydrophilic polyacrylamide (pAcDED) was obtained by polymerization of a novel acrylic monomer that resulted water soluble thanks to the short alkyl chain (ethyl chain) bearing the cationic amine group. Therefore, this polymer was employed as carrier for usnic acid.

The use of a chain transfer agent in the synthesis controlled polymer molecular weight, as demonstrated by the data reported in Table 1.

In the same table, DLS data show that pAcDED_T=0_ and pAcDED_T=0.5_ in water possessed a remarkable size of 920 and 500 nm, respectively. These values suggest that the polymer chains interact with each other, leading to large aggregates. In GPC analysis, these aggregation phenomena were avoided by adding NaCl in the eluent.

**Figure 1:**
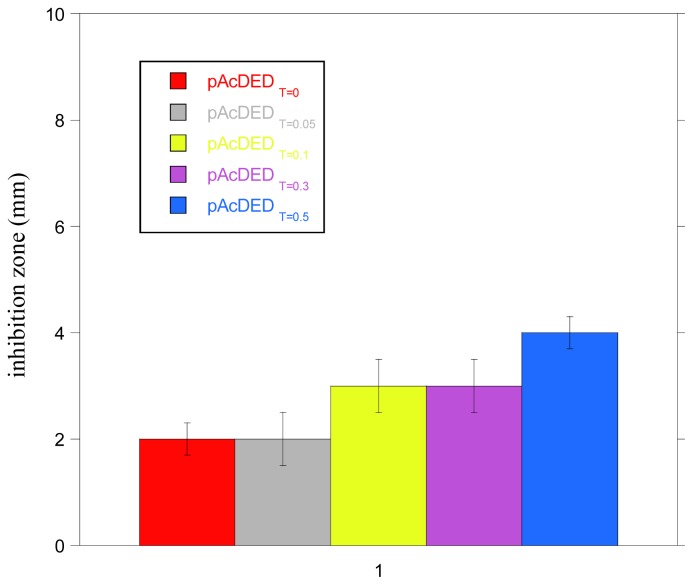
Inhibition zones of bacterial growth of polymers synthesized with different amount of chain transfer agent (T).

In Figure 1, the size of inhibition zone of bacterial growth for the polymers synthesized with different amounts of chain transfer agent is reported. As can be observed, all of polymers showed small inhibition zones, indicating a poor diffusion ability, probably due to their large molecular size and possible interaction with ionic components of the agar medium. The lowest molecular weight polymer (pAcDED_T=0.5_) displayed the largest inhibition zone (4 mm).

For all pAcDED polymers, a MIC value of 100 μg/mL was found. This minimum inhibitory concentration is comparable to those reported in literature for polymers based on ammonium salts [32].

**Figure 2:**
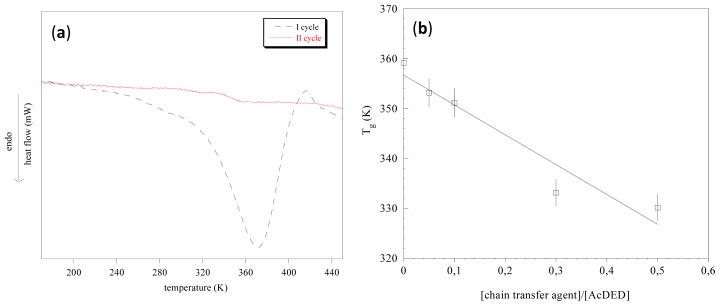
pAcDED_T=0_ thermograms related to the first and second heating cycle (**a**); dependence of polymer glass transition temperature (*T*_g_) on the chain transfer agent/[AcDED] ratio (**b**). The *T*_g_ values were determined by the first derivative of the curve obtained in the second heating cycle.

Differential scanning calorimetry evidenced that pAcDEDs were amorphous polymers. As an example, the thermogram of pAcDED_T=0_ is reported in Figure 2a. In the first scan, a wide endothermic peak related to the adsorbed water can be observed. This peak in the second scan disappeared. To evidence if any transition was present, the first derivative of the curves was done. A glass transition temperature at 359 K was found.

This transition remained unchanged in third scan. With increasing of the [chain transfer agent]/[AcDED] molar ratio employed in the synthesis, a decrease in glass transition temperature (Figure 2b) was observed, indicating a higher chain flexibility of the lower molecular weight pAcDEDs. This was presumably due to the higher concentration of the chain-ends.

**Figure 3:**
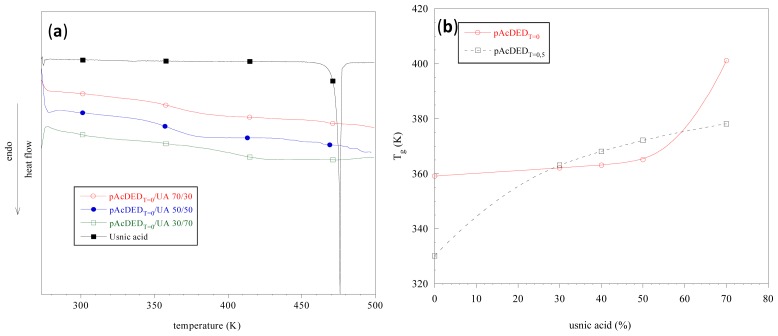
Differential scanning calorimetry (DSC) curves of usnic acid and pAcDED_T=0_/usnic acid (UA) complexes (**a**); dependence of polymer glass transition temperature on usnic acid content for pAcDED_T=0_ and pAcDED_T=0.5_ (**b**). The *T*_g_ values were determined by the first derivative of the curve obtained in the first heating cycle.

Differential scanning calorimetry (DSC) also allowed for the assessing of the establishment of polymer/drug interactions. In fact, the disappearance or shifting of exothermic or endothermic peaks, as well as the variation of enthalpy values can give information on the compatibility/incompatibility of the components in the formulation. In Figure 3a, DSC curves of UA (usnic acid), pAcDED_T=0_/UA 30/70, 50/50 and 70/30 are reported.

As can be observed, UA is a crystalline drug with a melting temperature of 476 K (melting enthalpy = 98 J/g). When UA was dispersed in pAcDED, the absence of the melting peak in all the tested formulations indicates that UA was in an amorphous state. This is probably due to the establishment of polymer/drug acidic-base interactions hindering UA crystallization. These interactions also caused a stiffening of polymer chains, as demonstrated by the increase in polymer *T*_g_ (Figure 3b). This stiffening occurs at a 30% UA content for the lower molecular weight polymer (pAcDED_T=0.5_) and only at high UA content (70%) for pAcDED_T=0_. Thus, due to its basic and amorphous features, pAcDED seems to be a good carrier for UA, since a molecular dispersion of the drug and a complete drug/polymer miscibility in the amorphous phase were achieved.

**Figure 4:**
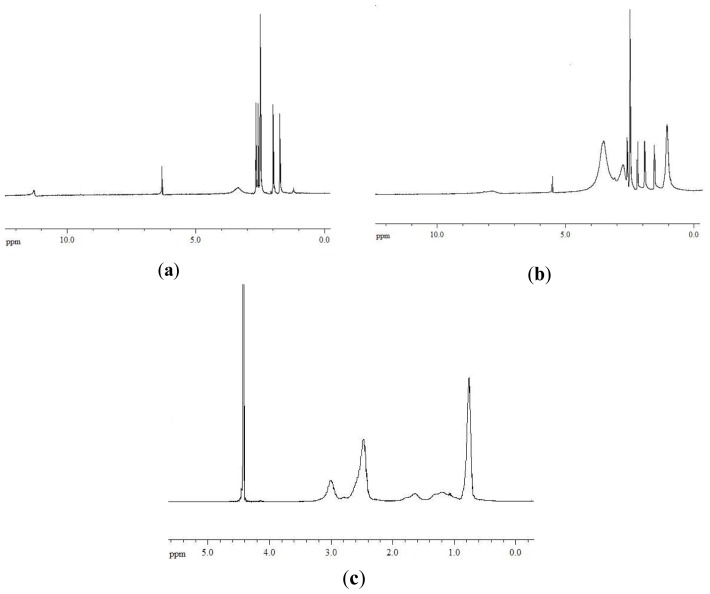
^1^H-NMR spectra of usnic acid (**a**), pAcDED_T=0_/UA 70/30 complex (**b**) and pAcDED_T=0_ (**c**).

A further confirmation of the polymer/drug interactions was given by ^1^H-NMR measurements. ^1^H-NMR spectra of usnic acid, pAcDED_T=0_/UA 70/30 complex (equiv_(basic group)_/equiv_(acidic group)_ ratio = 1/0.5) and pAcDED_T=0_, are reported in Figure 4a–c.

The UA spectrum showed a resonance at 11.3 ppm related to the protons of the hydroxyl groups in 3, 7 and 9 position (Figure 4a). The signal at 6.2 ppm was attributed to the proton in position 4, while the resonances at 2.65, 2.57, 1.98 and 1.71 ppm were attributed to the methyl groups in 12, 14, 15 and 10 position. In the spectrum of the pAcDED_T=0_/UA 70/30 complex, the disappearance of the signal at 11.3 ppm indicated the establishment of acid-base interactions between UA and pAcDED (Figure 4b). These interactions also caused shifts of the signals attributed to the CH in position 4 (from 6.2 to 5.4 ppm) and to the CH_3_ in position 12 (from 2.65 to 2.20 ppm). Similar results were found for the other complexes, where the equiv_(basic group)_/equiv_(acidic group)_ ratio was ≥1/1 (data not shown).

Also experiments of drug water dissolution showed the strong polymer/drug interactions. Indeed, differently from the free UA, a complete tablet dissolution was achieved after 30 min of immersion in water for all the tested formulations. This indicates that the polymer promotes UA solubility in water. However, since a significant variation of the UV spectrum was observed after tablet dissolution, it was not possible to determine the increase of UA solubility in water. In fact, other than the adsorption band of the free drug (290 nm), a new band, strongly dependent on the polymer/drug ratio, appeared at 240 nm (data not shown). This suggests the formation of a stable polymer/UA complex both at low and high drug concentrations.

As for the size of complexes in solution, DLS analysis showed a decrease in complex sizes with respect to those of polymers alone, due to the ability of usnic acid to shield polymer positive charges breaking the inter-chain interactions (Table 2). Indeed, the drug can establish multipoint interactions with polymer chains, promoting the formation of small structures.

Such a size decrease was higher for the polymers at lower molecular weight (pAcDED_T=0.1_ and pAcDED_T=0.5_) than the polymer obtained without a chain transfer agent. This finding can be attributed to the large initial size of pAcDED_T=0_ aggregates that did not allow UA to interact efficiently with the polymer cationic groups. The greater size of pAcDED_T=0_ with respect to other polymers is due to not only the higher length of polymer chains, but also, probably, to the lower concentration of chain ends. This involves a minor excluded volume, that allows each chain interacting with nearby chains.

Moreover, the complex size depended also on usnic acid content (Table 2). Generally, a slight increase of the complex size was observed with the increasing of the drug amount. The size of the pAcDED_T=0.1_/UA and pAcDED_T=0.5_/UA complexes could be suitable for a good cellular uptake [22].

To verify the effect of polymer charge density on the antibacterial properties of complexes, the pAcDED_T=0_/UA and pAcDED_T=0.5_/UA samples at different polymer/UA ratio were submitted to the disk diffusion tests. The pAcDED_T=0_/UA complexes showed a diameter of inhibition zone ranging from 4 mm for polymer/UA 70/30 to 2 mm for polymer/UA 30/70, values comparable to that of UA (2 mm). This finding indicates a poor diffusion ability of pAcDED_T=0_/UA complexes, due to their remarkable size (Table 2).

On the contrary, as shown in Table 3, the antibacterial activity of pAcDED_T=0.5_/UA complexes improved with respect to both UA and the polymer alone, particularly for the pAcDED_T=0.5_/UA 70/30. This result confirms that the inhibition zone depends mainly on the size of the complexes, rather than the drug amount. Therefore, UA, being strongly linked to the polymer, cannot directly act against bacteria, but can potentiate polymer activity by reducing the size of aggregates.

The broth microdilution assay was performed on UA, after its solubilization with dimethylsulfoxide (2% wt), pAcDEDs and on pAcDED_T=0.5_/UA complexes, these latter being chosen for the better antimicrobial activity showed in the disk diffusion tests. For all pAcDED polymers, a MIC value of 100 μg/mL was found. This minimum inhibitory concentration is comparable to those reported in literature for polymers based on ammonium salts [32]. In Table 3, the MIC values of UA, pAcDED_T=0.5_ and pAcDED_T=0.5_/UA complexes against *S. epidermidis* are reported. All pAcDED_T=0.5_/UA complexes possessed a lower MIC with respect to UA (*p* < 0.01).

Also in this case, the good activity of the complexes can be attributed to their reduced size. Indeed, the killing effect extent seems to be not significantly dependent on UA content in the samples (Table 3).

Then, the UA complexation with a cationic amorphous polymer at low molecular weight allowed not only a successful drug solubilization, but also an improvement of the polymer activity, thanks to the reduction of the aggregate size.

Since the small size of complexes can promote their penetration into the bacterial membrane, we suppose that there could be a combined action of the two antimicrobials. Indeed, the complexes into the cells should undergo enzymatic degradation, with consequent drug release and action.

In addition, since the best results were obtained for the complex at a low drug content (pAcDED_T=0.5_/UA 70/30), this strategy is particularly winning, given the known UA toxicity. Also the polymer toxicity could be reduced since the interaction with the drug partially neutralizes polymer positive charges. In addition, the combined use of two antimicrobial agents at different mechanism of action reducing the risk of emergence of drug-resistant microorganisms makes these systems particularly interesting for clinical application.

## 3. Experimental Methods

### 3.1. Monomer and Polymer Synthesis

Three acrylic monomers were synthesized by reaction between acryloyl chloride (Ac, Fluka, Buchs, Switzerland) and *N*,*N*-diethylethylenediamine (DED, Sigma Aldrich, St. Louis, MO, USA) or *N*,*N*-dipropylethylenediamine (PED, Santa Cruz Biotechnology Inc., Santa Cruz, CA, USA) or *N*,*N*-dibutylethylenediamine (BED, Fluka, Buchs, Switzerland). The obtained monomers were called AcDED, AcPED and AcBED, respectively. The synthesis procedure was: 0.029 mol of each diamine was added to a solution of Ac (0.038 mol) in dimethyl carbonate (DMC, 75 mL, Sigma Aldrich, St. Louis, MO, USA) containing K_2_HPO_4_ (0.08 moles, Carlo Erba, Milan, Italy), used as the base. The reaction was carried out for 4 h at room temperature. In all reactions, no side product was found. After reaction, the solution was filtered to remove the inorganic and organic salt, and the monomer recovered by solvent evaporation (yield ranging from 85% to 90%).

Differently from the AcDED monomer, AcPED and AcBED were not soluble in water. Since the object of our work was to develop water soluble antimicrobial polymers to be employed as carriers for hydrophobic drugs, only the AcDED monomer was used for radical polymerization.

**Scheme 1:**
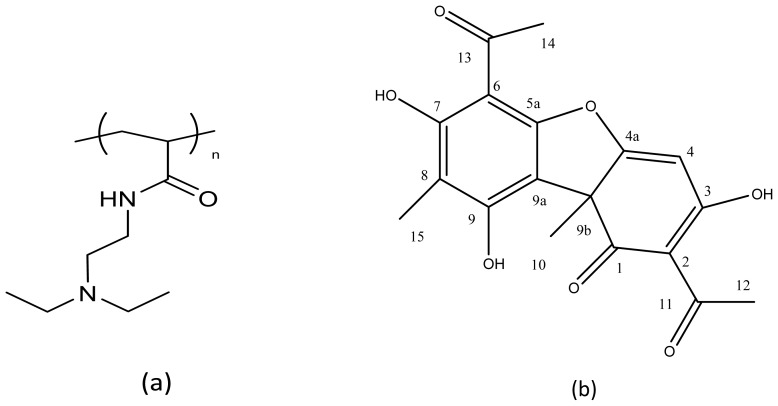
Structure formula of pAcDED (**a**) and usnic acid (**b**).

Polymer synthesis was carried out at 25 °C for 24 h by using a 1 M water solution of AcDED (5 mL) and K_2_S_2_O_8_ (2.8 × 10^−4^ mmol, Carlo Erba, Milan, Italy) plus FeSO_4_ (2.4 × 10^−4^ mmol, Carlo Erba, Milan, Italy) as radical initiators. The resulting polyacrylamide (Scheme 1a) was called pAcDED (pK_b_ = 8.61).

To evaluate the effect of polymer molecular weight on usnic acid dissolution in water, polymers at different chain length were obtained by adding during the synthesis sodium metabisulfite (Na_2_S_2_O_5_, Carlo Erba, Milan, Italy) as the chain transfer agent (T). The following chain transfer agent/monomer molar ratios were employed: [T]/[AcDED] = 0, 0.05, 0.1, 0.3 and 0.5. Resulting polymers were called pAcDED_T=0_, pAcDED_T=0.05_, pAcDED_T=0.1_, pAcDED_T=0.3_ and pAcDED_T=0.5_.

### 3.2. Polymer Characterization

^1^H-NMR spectra were performed employing a Varian XL 300 instrument and Chloroform (CDCl_3_) or water (D_2_O) as solvents. The obtainment of AcDED monomer and the respective polymer (Figure 4c) was confirmed by ^1^H-NMR (300MHz, CDCl_3_ and D_2_O): AcDED δ = 0.95 (t, *J* = 7.2 Hz, 6H, N(CH_2_C*H_3_*)_2_), 2.56–2.47 (m, 6H, C*H_2_*N(C*H_2_*CH_3_)_2_), 3.34–3.28 (m, CONHC*H_2_*), 5.52 (dd, *J*_1_ = 9.9 Hz, *J*_2_ = 2.1 Hz, 1H, C*H*H=CH), 6.07 (dd, *J*_1_ = 9.9 Hz, *J*_3_ = 16.8 Hz, 1H, CHH=C*H*), 5.52 (dd, *J*_3_ = 16.8 Hz, *J*_2_ = 2.1 Hz, 1H, CH*H*=CH), 6.70 (s, 1H, CON*H*); pAcDED_T=0_ δ = 0.76 (s, 6H, N(CH_2_C*H*_3_)_2_), 1.64–1.20 (m, 3H, C*H*_2_C*H*), 2.47 (s, 6H, C*H*_2_N(C*H*_2_CH_3_)_2_), 2.99 (s, 2H, CONHC*H_2_*).

Molecular weight measurements were carried out at 25 °C using a gel permeation chromatography (GPC) system equipped with a Shimadzu RID-10A differential refractive index detector and Tosoh TSK polyacrylamide gel columns. The calibration curve was obtained by using polyethylene oxide as the standard. Aqueous sample solutions containing 0.1 M NaCl were injected by an autosampler Hitachi AS-2000 at a flow rate of 0.8 mL/min.

pAcDED_T=0_ possessed a *M*_n_ of 120 × 10^3^ g/mol and 1.35 polydispersity index. By increasing the [T]/[AcDED] molar ratio, the polymer molecular weight decreased, reaching a *M*_n_ value of 69 × 10^3^ g/mol for a 0.5 molar ratio (Table 1).

The size of the polymers was evaluated by dynamic light scattering analysis (Coulter LS-13320) employing water solutions at a 0.1 mg/mL concentration.

Differential scanning calorimetry (DSC) measurements were carried out by a METTLER TA 3000 calorimeter provided with a TC 10 A processor by keeping the cell (DSC30) under N_2_ flow. The explored temperature range was 173–523 K, and the employed heating rate was 10 K/min. For each sample, two scans were performed.

### 3.3. Preparation of pAcDED/UA Complexes and Their Characterization

Polyacrylamide/usnic acid (UA, Sigma Aldrich,, St. Louis, MO, USA) (*M* = 344 g/mol, pKa_3_ = 4.4, pKa_9_ = 8.8, pKa_7_ = 10.7, Scheme 1b) complexes were prepared by dissolving in water the pAcDEDs, having different molecular weights, with the drug at the following polymer/UA weight ratios, 70/30, 60/40, 50/50 and 30/70. In particular, 50 mg of UA were added to 5 mL polymer solutions containing different amounts of polymer ranging from 116 to 22 mg.

Considering that pAcDEDs have one basic group per repetitive unit, while the drug has three acidic groups, the employed weight ratios correspond to equiv_(basic group)_/equiv_(acidic group)_ ratio of 1/0.5, 1/1, 1/2 and 1/3. After water evaporation under vacuum, powders were recovered and stored at 4 °C.

^1^H-NMR measurements were carried out, dissolving the complexes in dimethyl sulfoxide (DMSO-d_6_).

Thermal and DLS analyses were performed on the polymer/UA complexes by employing the same conditions used for the polymers.

To evaluate UA dissolution in water, tablets containing 50 mg of drug and a variable amount of pAcDEDs were prepared and kept in water up to complete dissolution. A tablet with only UA was prepared as the control. At a specified time frame, aliquots of the solutions were collected and tested by UV-spectroscopy (290 nm).

The antibacterial activity of samples was assessed against *Staphylococcus epidermidis* (ATCC 35984) by the disk diffusion test and broth microdilution assay. The former test was carried out on cellulose disks embedded with 20 μL of a 1 mg/mL polymer alone or polymer/UA solution and placed in Petri plates previously seeded with a 10^8^ CFU/mL bacterial concentration. Following incubation at 37 °C for 24 h, the diameters of inhibition zones of bacterial growth were measured.

The broth microdilution assay allowed instead to determine the minimum inhibitory concentration (MIC) of UA, pAcDEDs and polymer/UA complexes. As for UA, because of its limited water solubility, dimethylsulfoxide (2% wt) was used as a solvent mediator for the antimicrobial agent, after ruling out any intrinsic activity of dimethylsulfoxide by plating viability [6]. As for polymer/UA complexes, tablets containing a fixed amount of drug (500 μg) and variable amounts of polymer to obtain 70/30, 60/40, 50/50 or 30/70 pAcDED/UA weight ratios were prepared and dissolved in water (1 mL). Then, a series of dilutions was prepared and inoculated with a 10^6^ CFU/mL bacterial concentration. After 24 h-incubation at 37 °C, bacterial growth was determined by measuring the absorbance at 600 nm. All the experiments were done in triplicate. Analysis of variance was performed using MiniTab. Differences were considered significant for *p* < 0.05.

## 4. Conclusions

To increase the bioavailability of water insoluble drugs, novel basic polyacrylamides (pAcDEDs) at different molecular weights were synthesized and used as carriers. All obtained polymers were amorphous, water-soluble and able to complex usnic acid, chosen as the model drug.

DSC and ^1^H-NMR measurements showed that the polymers established multipoint acid-base interactions with the drug phenolic groups. The shielding of polymer positive charges by usnic acid allowed the obtainment of polymer/UA complexes smaller in size than the polymers alone, particularly for polymers with a lower molecular weight. The drug-polymer multipoint interactions were more favored in the polymers with a higher concentration of chain ends. Indeed, a complex size of about 60 nm was obtained with the lowest molecular weight polymer at a low usnic acid content (pAcDED_T=0.5_/UA 70/30).

The polymer molecular weight affected also the antimicrobial activity against *S. epidermidis* of both the polymer alone and polymer/UA complexes. Indeed, pAcDED_T=0.5_ displayed the largest inhibition zone of bacterial growth, both alone and when combined with UA.

The good activity of the complexes is probably due to their reduced size, which allows an efficient cellular uptake of the antimicrobial complexes. The killing effect extent seems to be not significantly dependent on UA content in the samples.

Novel polymers here reported could be used as a carrier for different types of molecules (drugs, DNA, vaccines) that similarly to usnic acid, have toxicity issues or need a targeted release. Further investigations are needed to determine whether this complexation allows a safer use of the cationic polymer and usnic acid in therapeutics.

## Figures and Tables

**Table 1:** Molecular weight, polydispersity index and dynamic light scattering (DLS) size of synthesized polymers.

POLYMER	*M*_n_ (10^3^ g/mol)	*I* = *M*_w_/*M*_n_	Size (nm) *
pAcDED_T=0_	120	1.35	920 ± 40
pAcDED_T=0.05_	100	1.30	880 ± 80
pAcDED_T=0.1_	94	1.32	780 ± 50
pAcDED_T=0.3_	78	1.29	680 ± 70
pAcDED_T=0.5_	69	1.31	500 ± 70

* The data, representative of at least six measurements, are reported as the arithmetic mean ± standard deviation.

**Table 2:** DLS size of polymer/UA complexes.

pAcDED/UA	Size (nm)		

	[T]/[AcDED] = 0	[T]/[AcDED] = 0.1	[T]/[AcDED] = 0.5
100/0	920 ± 40	780 ± 50	500 ± 70
70/30	530 ± 40	70 ± 7	60 ± 10
60/40	600 ± 30	90 ± 8	80 ± 10
50/50	580 ± 50	95 ± 15	90 ± 9
30/70	610 ± 70	100 ± 15	100 ± 10

The data, representative of at least six measurements, are reported as the arithmetic mean ± standard deviation.

**Table 3:** Inhibition zones of bacterial growth and minimum inhibitory concentrations (MICs) of usnic acid, pAcDED_T=0.5_ and pAcDED_T=0.5_/UA complexes.

Sample	Inhibition Zone (mm)	MIC (μg/mL)
Usnic acid	2 ± 0.5	16 ± 4
pAcDED_T=0.5_	4 ± 1	100 ± 3
pAcDED_T=0.5_/UA 70/30	15 ± 2	5 ± 1
pAcDED_T=0.5_/UA 60/40	9 ± 1	7 ± 1
pAcDED_T=0.5_/UA 50/50	9 ± 2	8 ± 1
pAcDED_T=0.5_/UA 30/70	9 ± 2	10 ± 2
